# Dermatology Journals’ Editorial Boards Require Improved Gender Equity: JMIR Dermatology’s Future Directions

**DOI:** 10.2196/43256

**Published:** 2023-05-05

**Authors:** Sarah Minion, Julianne Kiene, Robert Dellavalle

**Affiliations:** 1 Carver College of Medicine University of Iowa Iowa City, IA United States; 2 Georgetown University School of Medicine Washington, DC United States; 3 Department of Dermatology University of Colorado School of Medicine Aurora, CO United States

**Keywords:** gender equity, dermatology, editorial board members, diversity, gender, inclusion, equity, academia, equality

## Abstract

Gender disparities exist across all facets of academic medicine including within the editorial boards of dermatology journals. Only 22% of these editorial boards comprised women, even though 51% of full-time, faculty dermatologists are female. When inviting academic dermatologists to our editorial board at *JMIR Dermatology*, we invited 50% women to represent the gender distribution of academic dermatologists; however, we have not sufficiently reached gender equity among accepted editorial board members. We will continue to strive toward the goal of gender equity on our editorial board and invite other dermatology journals to do the same.

Although roughly equivalent rates of women and men have applied, been accepted to, and graduated from medical school since the early 2000s, gender disparities persist in academic medicine. In dermatology specifically, the percentage of female medical school graduates and residents did not reach 50% until the 1990s [1,2]. The gender discrepancy is seen in many facets of academia including lower rates of publishing, first-author publications, receiving National Institutes of Health grants, serving as journal editors, and being included on journal editorial boards [3-7].

Women constitute a minority in dermatology journal editorial boards. One review study of all dermatology journals found that only 22% of editorial board members were women [8]. Another recent analysis exploring gender parity of the top 20 dermatology journals’ editorial boards found that women comprised a median of 33.4% (IQR 27.2%-46.9%) of their editorial boards [9]. Only in 4 of the 20 top journals did women comprise a >50% average of the editorial board [9], even though a 2018 report by the Association of American Medical Colleges found that 51% of full-time, faculty dermatologists are women [10]. Among the top 20 journals, 75% had a male editor-in-chief [9] and 81% of all dermatology journals had a male editor-in-chief [8].

At *JMIR Dermatology*, our goal was to represent the gender distribution of academic dermatologists by inviting 50% women to join our editorial board. After recruitment and acceptance, we found that the gender distribution of our editorial board closely resembles that of the top 20 dermatology journals, with only 8 (32%) out of 25 board members being women. Although our goal was to create an equitable editorial board, *JMIR Dermatology* has fallen short of our 50% target. Based on our in-house review, it will be necessary to invite more than 50% female dermatologists to achieve our gender equity goal.

Although gender equity in academia is a widely discussed topic, gender disparities persist across all facets of academic medicine. The specialty of dermatology is no exception. Improving equity in certain parts of academia could start with improving the equitable distribution of editorial board membership; yet, a majority of the top dermatology journals today have failed to reach these goals. We at *JMIR Dermatology* will continue to work toward gender equity on our editorial board. Further steps for our journal may include inviting inequitable proportions of female dermatologists until we reach our goal or investigating the reasons behind why there was lower acceptance among applications from women. We hope our generation and future generations of dermatologists will continue to work toward the goal of gender equity in academia.
